# Norms for Automatic Estimation of White Matter Hyperintensities Burden: LST-AI Service in neuGRID

**DOI:** 10.3390/diagnostics16152460

**Published:** 2026-08-04

**Authors:** Alberto Boccali, Silvia De Francesco, Claudio Crema, Claudio Demaria, Cesare M. Baronio, Damiano Archetti, Alberto Redolfi

**Affiliations:** Laboratory of Neuroinformatics, Istituto di Ricovero e Cura a Carattere Scientifico Istituto Centro San Giovanni di Dio Fatebenefratelli, via Pilastroni 4, 25125 Brescia, Italy; aboccali@fatebenefratelli.eu (A.B.); ccrema@fatebenefratelli.eu (C.C.);

**Keywords:** white matter hyperintensity, norms, LST-AI, deep learning, neuroimaging

## Abstract

**Background**: White Matter Hyperintensities (WMH) are common MRI markers of cerebral small-vessel disease and are associated with cognitive impairment and dementia. Deep Learning (DL) tools have improved WMH segmentation, enabling faster and more reproducible lesion quantification. However, the lack of normative reference frameworks limits the clinical and translational use of WMH volumes. Aims: to develop and validate normative reference curves for automated WMH quantification and to define clinically useful thresholds for rule-out and rule-in interpretation in memory-clinic settings. **Methods**: We developed age- and sex-adjusted WMH normative models for 2D (*n* = 788) and 3D (*n* = 895) FLAIR acquisitions in cognitively normal individuals aged 40–95 years. WMH volumes were segmented using LST-AI and normalized to total intracranial volume. Normative percentiles were derived using Generalized Additive Models for Location, Scale and Shape (GAMLSS) with a Johnson’s SU distribution and externally validated in 458 individuals spanning cognitively normal (CN), mild cognitive impairment (MCI), and dementia groups from two validation cohorts. Normative distributions have been made available through neuGRID, an online platform providing AI-based tools for neuroimaging analysis. **Results**: WMH burden increased progressively with age in both normative datasets and showed a stepwise increase across the cognitive continuum from CN to MCI and dementia. The optimal balanced thresholds corresponded to the 92nd percentile for the 2D model and the 85th percentile for the 3D model, yielding areas under the receiver operating characteristic curve (ROC-AUCs) of 0.71 (95% CI: 0.63–0.78) and 0.68 (95% CI: 0.61–0.74), respectively. Secondary threshold analyses highlighted complementary operating characteristics, with the 2D model favoring sensitivity and the 3D model favoring specificity, supporting their potential use in sequential diagnostic workflows. **Conclusion**: This study provides an externally validated normative framework for interpreting LST-AI-derived WMH burden through the neuGRID single-case service. The proposed modality-specific norms enable standardized identification and contextualization of elevated WMH burden and may support clinical stratification, second-opinion assessment, and research applications in cognitive disorders.

## 1. Introduction

Neuroimaging research has traditionally focused on brain atrophy as a major marker of neurodegeneration. Increasing evidence, however, suggests that White Matter Lesions (WML) may also contribute to cognitive impairment and dementia through mechanisms that are not exclusively attributable to vascular pathology [1]. On Magnetic Resonance Imaging (MRI), these lesions appear as White Matter Hyperintensities (WMH) on T2-weighted and fluid-attenuated inversion recovery (FLAIR) sequences and have historically been regarded as markers of cerebral small-vessel disease. Neuropathological studies indicate that WMH, particularly in Alzheimer’s disease (AD), may arise from multiple mechanisms, including chronic ischemic injury related to arteriolosclerosis and axonal degeneration secondary to cortical AD pathology associated with amyloid-β and hyperphosphorylated tau deposition [1,2,3]. Furthermore, regional differences in WMH etiology have been reported, with parietal and temporal lesions appearing more strongly associated with neurodegenerative processes, whereas frontal lesions may reflect a combination of vascular and degenerative mechanisms [1].

Neuroimaging studies have consistently demonstrated that WMH burden increases across the AD continuum, in both dominantly inherited and sporadic forms of the disease, and that this association may be partially independent of traditional vascular risk factors [4]. Large cohort studies have reported substantially greater WMH volumes in individuals with AD compared with cognitively normal (CN) older adults and have linked increasing WMH burden to a higher risk of dementia [5]. Collectively, these findings suggest that WMH reflect relevant pathological processes associated with cognitive decline and neurodegeneration, in addition to vascular injury [6].

In routine clinical practice, WMH burden is commonly evaluated using visual rating scales such as Fazekas [7] and Wahlund [8]. Although widely adopted, these scales rely on subjective interpretation and provide only coarse ordinal estimates of lesion burden. Their use therefore remains vulnerable to inter-rater variability and limited sensitivity to subtle changes, particularly in individuals with low lesion burden. Moreover, visual ratings are not ideally suited for harmonized quantitative analyses across scanners, protocols, and research cohorts. These limitations have stimulated increasing interest in automated and standardized WMH quantification.

Automated WML segmentation methods have evolved substantially over the past decade. Early approaches, including the Lesion Segmentation Tool (LST), relied on engineered image-processing pipelines [9]. More recently, artificial intelligence (AI) and deep learning (DL) techniques have improved segmentation accuracy and robustness. Among these approaches, LST-AI represents a DL-based extension of LST built on an ensemble of three 3D U-Net architectures and specifically designed to improve WMH detection, including small lesions that are frequently missed by conventional methods [10]. Comparative evaluations have demonstrated competitive performance relative to established segmentation frameworks and other DL-based approaches [10,11].

Despite these advances in WMH quantification, an important translational gap remains. While automated tools can provide accurate lesion volumes, there is currently no widely available normative framework for interpreting DL-derived WMH measurements at the individual subject level. Existing neuroimaging norms have been developed for markers such as WML volume, ventricular enlargement, and hippocampal atrophy [12,13], but these frameworks were generally derived using traditional quantification methods rather than modern pipelines. Consequently, clinicians and researchers lack standardized references for determining whether a WMH burden is unusually high for a given individual, comparing results across studies, or interpreting measurements generated by different segmentation approaches.

The present study addresses this gap by developing age- and sex-adjusted normative models for quantitative WMH data computed with LST-AI for both 2D and 3D FLAIR acquisitions. Specifically, we aim to: (1) derive modality-specific normative percentiles and operational thresholds for WMH burden; (2) validate these norms in independent cohorts spanning cognitively normal individuals, mild cognitive impairment (MCI), and dementia; (3) evaluate whether increasing WMH burden is associated with progression along the cognitive continuum; and (4) deploy the resulting tool as a freely accessible online service for the research and clinical communities via neuGRID platform (https://neugrid2.eu). By providing normative standards for DL-derived WMH quantification, our work aims to facilitate a standardized interpretation of WMH burden and support research and clinical applications in cognitive disorders.

## 2. Materials and Methods

### 2.1. Study Design

Normative percentiles were created based on WMH total volumes quantified by LST-AI processing of 3D or 2D FLAIR images. The 3D normative cohort comprised clinically defined CN drawn from multiple validation datasets: ADNI (*n* = 718) [14], PPMI (*n* = 31) [15], NIFD (*n* = 110) [16], EDSD (*n* = 10) [17], and OASIS (*n* = 26) [18]. The 2D normative cohort consisted of control individuals from EDSD (*n* = 81), OASIS (*n* = 511), and ARWIBO (*n* = 196) [19]. Both norms were validated using validation external datasets. The NACC cohort was used for validation of the 3D norms [20], while the MCSA cohort was used for validation of the 2D norms [21]. For each validation dataset, participants were stratified into three diagnostic categories: CN, MCI, according to Petersen criteria [22], and AD, according to the 2011 NIAA criteria [23] for NACC, while CN, MCI, and Dementia disease (DM) cases in MCSA were defined according to a centralized Mayo Clinic consensus panel of experts [21]. The service we developed (pipeline and percentiles) has been made available through neuGRID (https://neugrid2.eu), an online platform that provides tools for automated quantification of core biomarkers for neurodegenerative diseases [24]. Supplementary Figures S1 and S2 show the neuGRID platform interface and the report sent to the end-user’s mailbox. The average time to obtain the LST-AI report, which highlights where the subject’s WMH total volume lies within the percentile distributions, is 1.5 h.

### 2.2. Data

The total number of individuals used for the 3D normative dataset was 895 CN, including 544 females. Similarly, the 2D normative dataset comprises 788 CN, including 453 females. The MRI data were collected from publicly available datasets hosted on the IDA platform (ADNI, PPMI, NIFD), as well as from the neuGRID infrastructure (EDSD and ARWIBO) and OASIS (https://sites.wustl.edu/oasisbrains). Cardiovascular risk factors were not used as exclusion criteria for the cognitively normal reference cohorts. Patient selection was based only on cognitive and neurological status rather than on the presence of vascular comorbidities, in order to increase the representativeness of our norms with respect to real-world aging populations. For the validation cohorts, NACC included 90 CN, 95 MCI, and 95 AD participants. MCSA data included 100 CN, 39 MCI, and 39 DM participants. The two validation datasets were identified via the GAAIN platform [25]. The imaging data used to generate the 3D and 2D norms were acquired from three main scanner manufacturers (Siemens—Munich (Germany), GE—Chicago (USA), Philips—Amsterdam (The Netherlands)) at both 1.5T and 3T. In the NACC validation cohort, imaging data were acquired on Siemens, GE, and Philips scanners, all at 3T. In the MCSA validation cohort, all images were acquired on a GE scanner at 3T.

### 2.3. White Matter Hyperintensities Volume Processing

WMH were segmented using LST-AI, a DL-based extension of LST comprising an ensemble of three 3D U-Nets [10,26]. Each U-Net architecture is based on nnUNet, a DL-based segmentation method that automatically configures itself [27]. Each 3D U-Net generates a lesion probability map. The mean of the three outputs is calculated and thresholded to produce the final binary lesion map. The imaging analysis pipeline used in our work involved providing LST-AI with native-space T13D and FLAIR images for each subject. LST-AI enables lesion segmentation across four distinct brain regions: infratentorial, juxtacortical, subcortical, and periventricular. Total lesion volume was calculated as the sum of lesion volumes in these four anatomical subregions. All lesion volumes extracted in native space were normalized to total intracranial volume (TIV), estimated using the Statistical Parametric Mapping (SPM12) toolbox. Normalization followed a standard procedure, dividing lesion volumes by the subject’s TIV and multiplying the result by a reference volume of 1409 mL to account for inter-individual differences in head size [28]. No post hoc scanner harmonization procedure was applied to WMH volumes. All processed outputs underwent systematic visual quality control performed by two experienced neuroscientists (AB, AR), who reviewed lesion masks and segmentation quality slice-by-slice. Blinding to diagnostic status was not implemented for the technical QC step, which focused exclusively on segmentation integrity and image quality. Scans with major segmentation failures or insufficient image quality were excluded. The final rejection rate was 170/1065 (15.9%) for the 3D normative dataset, 123/911 (13.5%) for the 2D normative dataset, 20/300 (6.7%) for the 3D validation dataset and 14/192 (7.3%) for the 2D validation dataset.

### 2.4. Demographics and Cognitive Variables

To characterize the validation cohorts, demographic information including education and ethnicity, as well as the main clinical neuropsychological test assessments, were collected. For NACC, the following cognitive measures were included: Montreal Cognitive Assessment (MoCA) to assess global cognitive functioning [29], Clinical Dementia Rating-Sum of Boxes (CDR-SB) to assess the overall dementia severity [30], Trail Making Test Part A (TMT-A) to evaluate processing speed and attention, and Trail Making Test Part B (TMT-B) for executive function including cognitive flexibility and set-shifting [31]. For MCSA, the battery included: Mini-Mental State Examination (MMSE), which provides a measure of cognitive status and assesses orientation, memory, language, and basic visuospatial abilities [32], and CDR-SB and global Clinical Dementia Rating (CDR), which assesses functional and cognitive impairment severity across multiple domains, including: memory, orientation, judgment, community affairs, and personal care [33].

### 2.5. Cut-Off Definition and Evaluation Metrics

To assess the alignment of the normative framework in the external validation cohorts, Z-estimated values were examined and summarized using the mean and standard deviation. Z-estimated values were calculated by comparing each participant’s normalized WMH volume from MSCA and NACC with the corresponding age- and sex-adjusted 2D or 3D normative reference distribution derived from CN, so that positive values indicate WMH burden above the expected norm for that modality.

To determine an operational threshold for distinguishing CN versus impaired participants (MCI, DM and AD), we conducted a ROC-based diagnostic accuracy analysis on the continuous normative Z-score. The primary cut-point was identified using the standard Youden index, which maximizes the joint performance of sensitivity and specificity. In addition, weighted Youden analyses were performed as a secondary, clinically oriented procedure to explore alternative operating points optimized for sequential diagnostic use.

The AUC-ROC curve was used as the primary measure of discriminative performance. For clinical interpretability, each selected threshold was also expressed as its corresponding normative percentile. Positive predictive value (PPV), negative predictive value (NPV), positive likelihood ratio (LR+), and negative likelihood ratio (LR−) were calculated to characterize the potential rule-in and rule-out utility of each threshold. High NPV combined with low LR− supports exclusion of clinically relevant WMH burden, whereas high PPV combined with high LR+ supports confirmation of elevated WMH burden. Because predictive values are prevalence-dependent measures, PPV and NPV were interpreted in the context of the external validation cohorts, which reflected secondary and tertiary memory-clinic populations rather than community-based samples.

### 2.6. Statistical Analysis

#### 2.6.1. Comparison Between Cohorts

Statistical analyses were conducted to assess demographic, clinical, and volumetric differences among datasets and subgroups.

For the normative cohorts, between the 3D and 2D populations, comparisons were performed using the non-parametric Mann–Whitney U test. To account for multiple comparisons, the false discovery rate was controlled using the Benjamini–Hochberg (FDR-BH) procedure. Categorical variables were compared between groups using the chi-square (χ^2^) test of independence based on contingency tables. The explained variance score for age, gender, education, ethnicity, vendor, and acquisition parameters (i.e., field strength, TE, TR, TI, flip angle, slice thickness) was calculated using quantile regression (Supplementary Table S1). For a more detailed comparison between 2D and 3D norms, effect sizes were standardized to a common metric ranging from 0 (no association) to 1 (perfect association) by transforming Cohen’s d values into r-indices for continuous outcomes, enabling direct comparability with Cramér’s V for categorical outcomes [34]. We estimated typical subject profiles at different ages to see how 2D and 3D normative data compare over time (see Supplementary Table S2a–d).

For the validation cohorts, differences across diagnostic groups were assessed using the non-parametric Kruskal–Wallis test, with pairwise post hoc comparisons using Dunn’s test. As before, categorical variables were compared across diagnostic categories using the χ^2^ test. Post hoc pairwise comparisons between diagnostic groups were also performed.

Then, all group comparisons (CN, MCI, AD, DM) were evaluated using a multivariate median quantile regression model, with clinical and volumetric models adjusted for age, sex, ethnicity, and educational attainment. Adjusted group-specific median estimates were then used for pairwise post hoc contrasts, and multiplicity was controlled using the FDR-BH procedure. Statistical significance was set at *p* < 0.05.

Receiver operating characteristic (ROC) analyses were performed to assess the ability of the normative models to discriminate between groups. The area under the ROC curve (AUC) was computed as a measure of discriminative performance, and diagnostic accuracy was evaluated separately for the 2D and 3D normative models. The primary cut-off was derived using the standard Youden index. In addition, weighted Youden analyses were conducted using a grid-search procedure to explore clinically oriented thresholds with different emphasis on sensitivity or specificity, depending on the intended sequential workflow (rule-out for the 2D model and rule-in for the 3D model). For each candidate threshold, sensitivity, specificity, PPV, NPV, LR+, and LR− were computed.

Statistical analyses were conducted in Python (version 3.11.9) using SciPy (version 1.13.1) and Statsmodels (0.14.4).

#### 2.6.2. Percentile Creation

Age and sex were selected as covariates for inclusion in the final normative model and percentile curves were derived conditionally on these demographic covariates. Consequently, the resulting norms are demographic-adjusted within each modality-specific reference system and should not be considered interchangeable across 2D and 3D acquisition protocols. Lesion volume trajectories were modeled as a smooth non-linear function using penalized B-splines. Percentile reference curves were generated using the “Generalized Additive Models for Location, Scale and Shape” (GAMLSS) framework [35] in R (version 4.4.2). To identify the most appropriate statistical distribution for modeling lesion volume data, multiple candidate distributions were evaluated. Because WMH burden is known to increase with age in cognitively normal populations, an increasing monotonic age trajectory was imposed during model fitting. Goodness of fit was assessed using the AIC/BIC information criterion. Johnson’s SU (JSU) distribution was selected as the best-fitting model for the observed data.

## 3. Results

### 3.1. Comparison Between 2D and 3D Normative

Table 1 summarizes the characteristics of the reference populations used to create the 2D and 3D norms. The two normative datasets should be interpreted as modality-specific reference frameworks rather than directly interchangeable norms, as the largest differences between cohorts were observed in acquisition-related variables, all of which showed large effect sizes. By contrast, demographic differences were comparatively modest, with small effects for ethnicity, age, and education, although the 3D cohort was on average older and more educated than the 2D cohort. WMH volumetric measures showed small effect sizes for periventricular, subcortical, and total lesion volumes, and negligible differences for infratentorial and juxtacortical volumes. Overall, these findings indicate that the limited comparability between the 2D and 3D normative models was driven primarily by differences in imaging protocol rather than by major differences in demographic composition or overall WMH burden. This interpretation is also consistent with the supplementary adjusted analyses, which showed no significant difference in adjusted median total WMH volume between the two normative groups.

### 3.2. Percentile Creation

**Figure 1:**
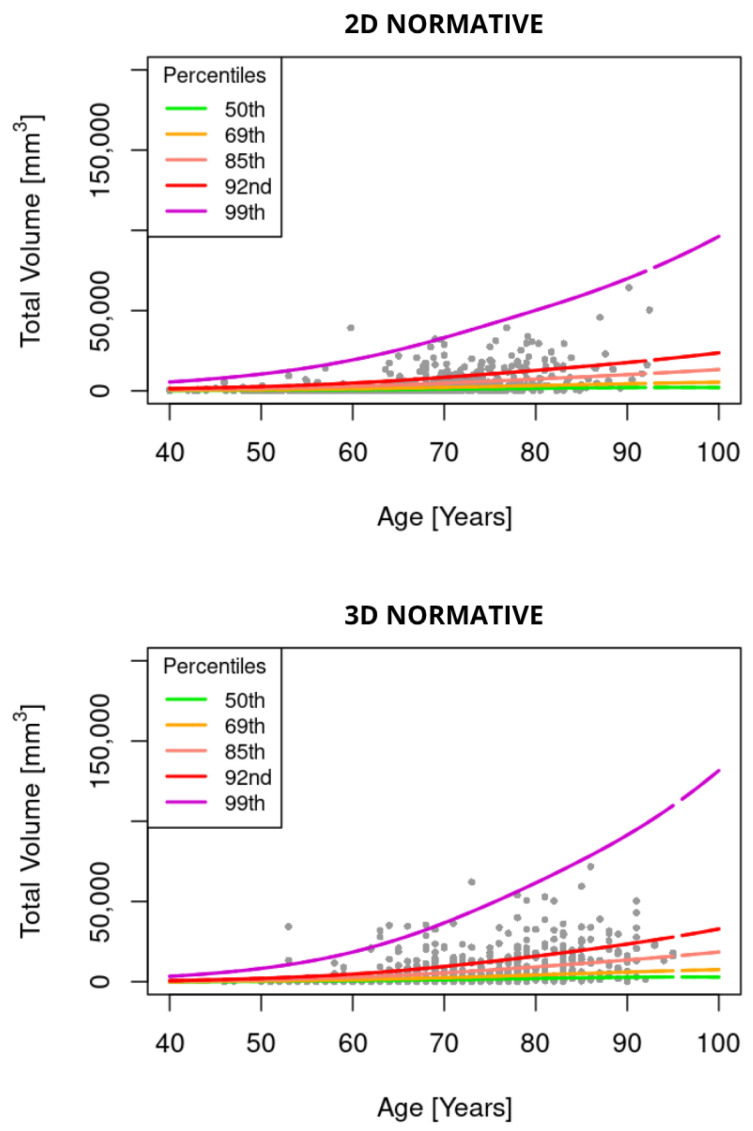
Age-specific percentile distribution of WMH adjusted for age and sex and normalized for total intracranial volume (TIV) in the 2D (**top**) and 3D (**bottom**) datasets. Volumes were estimated using LST-AI, and fitted percentile curves were derived using a Generalized Additive Models for Location, Scale, and Shape (GAMLSS) framework with a Johnson’s SU (JSU) distribution.

Figure 1 shows the percentile distribution derived from the 2D and 3D reference populations as a function of age and sex included as a covariate in the GAMLSS model. In both normative cohorts, total lesion volume increases with age. Multiple percentiles were derived (i.e., 50th, 69th, 85th, 92nd, 99th) for both normative groups to support individual-level quantification of WMH burden. These percentile distributions formed the basis for subsequent Z-score estimation and threshold analyses in the external validation cohorts.

### 3.3. Norms Validation

#### 3.3.1. Validation Cohorts’ Characteristics

Table 2 and Table 3 summarize the characteristics of the independent validation datasets, stratified by diagnostic categories. The 2D MSCA validation cohort showed typical clinical stratification across diagnostic groups, with older age and lower education in the DM group, together with significant differences in ethnicity. All images were acquired on the same 3T GE scanner using a standardized 2D FLAIR protocol, minimizing acquisition-related variability. Clinical severity increased progressively from CN to MCI to DM, as indicated by CDR-SB, CDR, and MMSE scores. Total WMH burden also increased significantly across groups, with the greatest contribution from periventricular lesions, suggesting that WMH accumulation in this cohort is primarily associated with disease severity rather than widespread changes across all other white matter compartments. Notably, the combination of a clinically heterogeneous sample and a technically homogeneous acquisition protocol provides an appropriate setting to test the robustness of automated WMH quantification under realistic yet controlled conditions.

In the 3D NACC, a different validation cohort scenario was observed, with no significant differences across groups in age, education, or ethnicity, and all individuals were scanned on 3T scanners. As expected in a multicenter dataset, scanner-related acquisition parameters showed some between-group variability, particularly for scanner manufacturer, flip angle, and slice thickness. Clinical and cognitive measures showed the expected stepwise worsening from CN to MCI to AD, supporting the clinical validity of the sample. Similarly, total WMH burden increased across diagnostic groups, consistent with a progressive accumulation of white matter damage along the disease continuum and reinforcing the relevance of WMH quantification in dementia-related phenotyping.

The heterogeneity of the two validation cohorts is a strength, as it tests the robustness of the norms across real-world acquisition settings. In the percentile-based analysis (Supplementary Table S3), the 2D validation cohort showed a progressive shift towards higher WMH percentile ranges across diagnostic groups, although percentile values were broadly elevated across all groups, consistent with the advanced age and disease burden of this sample. Infratentorial, subcortical, and periventricular regions showed less stable discrimination than the total brain area, suggesting lower sensitivity for diagnostic stratification. The 3D validation cohort confirmed the same overall direction, with the most pronounced separation observed in the periventricular and total regions. The total WMH percentile showed the greatest consistency across the 2D and 3D groups and therefore represents the most robust candidate for clinical interpretation.

#### 3.3.2. Diagnostic Metrics

Inspection of the Z-estimated distributions showed broader separation across diagnostic groups for the 2D normative model than for the 3D model. In the 2D validation cohort, the CN group had a mean Z-estimated value of 0.99 ± 0.75, followed by a progressive increase in MCI and DM; whereas in the 3D cohort the CN group was more closely centered around the expected normative mean (−0.08 ± 0.97), with a similar although less marked increase across MCI and AD. These findings suggest that the 2D model provided stronger group discrimination, whereas the 3D model showed better calibration in CN.

**Figure 2:**
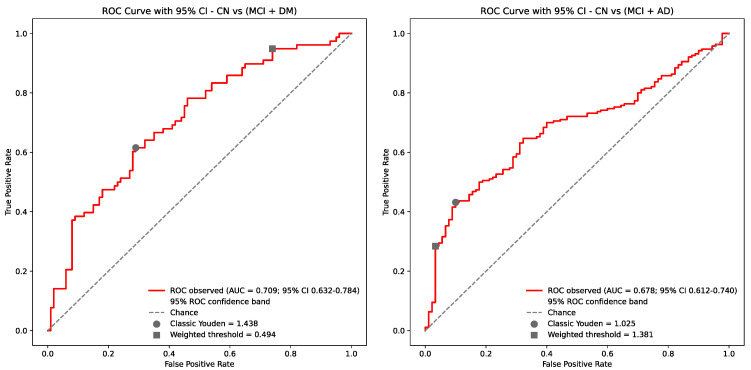
ROC analysis of GAMLSS-derived Z-scores based on Johnson SU (JSU) fitted normative distributions of LST-AI WMH volumes. The 2D (**left**) and 3D (**right**) models were evaluated for their ability to distinguish cognitively normal individuals from pathological participants (MCI, DM and AD).

Figure 2 summarizes the ROC analyses performed to assess the ability of normative models to discriminate CN from pathological individuals (MCI, DM, AD). The 2D FLAIR normative model showed slightly better overall discrimination than the 3D model for distinguishing CN from pathological individuals (MCI and DM).

In the primary balanced ROC analysis, the 2D model yielded an optimal threshold of 1.438 (92nd percentile), with sensitivity 0.62 and specificity 0.71, whereas the 3D model yielded an optimal threshold of 1.025 (85th percentile), with sensitivity 0.43 and specificity 0.90. The fact that both optimal thresholds fell within the upper tail of the distributions (85th-92nd percentile) supports the clinical relevance of a high WMH burden as a marker of pathological status (Table 4). These balanced thresholds were retained as the primary modality-specific reference cut-offs within the LST-AI neuGRID pipeline.

In a secondary clinically oriented analysis, based on weighted Youden optimization, the 2D threshold decreased to 0.494 (69th percentile), increasing sensitivity to 0.95 and reducing LR− to 0.20; while the 3D threshold increased to 1.381 (92nd percentile), raising specificity to 0.97, PPV to 0.95, and LR+ to 8.53 (Table 4). Therefore, the 2D model may be more appropriate for sensitivity-oriented case exclusion, whereas the 3D model may be better suited for specificity-oriented confirmation of elevated WMH burden.

## 4. Discussion

This study demonstrates that automated WMH quantification via LST-AI [10] can be translated into a clinically useful normative service within the neuGRID platform [24]. This work provides evidence that automated WMH quantification can capture the progressive increase in lesion burden across the cognitive continuum, delivering operational clinical thresholds. A central finding is that WMH burden increased with age in both normative datasets, confirming the expected age-related accumulation of lesion load [36] and supporting the biological plausibility of the fitted curves. Our norms are consistent with previous studies reporting an age-related increase in WMH load, with a steeper increment typically observed around 50 years of age [37,38]. The use of GAMLSS with a Johnson SU distribution enabled us to model the strongly skewed lesion volume distributions while preserving the non-linear age dependence of WMH burden [37,39]. This is an important methodological advantage because WMH volumes are not well described by simple linear or Gaussian assumptions, particularly in population samples including both WMH low-burden and high-burden individuals. In this sense, the derived percentile curves provide a more realistic representation of burden across the adult lifespan than raw volumes alone. Moreover, WMH volumes show substantial interindividual variability, and normalization to total intracranial volume improves comparability across individuals by accounting for head-size differences [40,41]. Sex was also modeled directly within the GAMLSS framework, further strengthening the clinical interpretability of the resulting norms.

The MCSA and NACC validation analyses further support the clinical relevance of the proposed framework. In both validation cohorts, WMH burden increased across the diagnostic continuum, with the expected worsening of clinical and cognitive measures from cognitively normal individuals to MCI and dementia [42,43]. This pattern was accompanied by a progressive shift towards higher percentile ranks, especially for total lesion burden and, to a lesser extent, for periventricular WMH [37]. These findings support the hypothesis that WMH are associated not only with aging, but also with neurodegenerative pathology, and therefore may represent a marker of disease severity [44,45]. These findings suggest that the percentile-based framework is sensitive to disease-related WMH accumulation and that the normative data capture more than simple age effects [38]. Importantly, the gradients were reproduced in two cohorts with different acquisition settings, strengthening confidence in the robustness of the approach under realistic multicenter conditions.

Among regional measures, total WMH burden emerged as the most stable and clinically useful indicator, although periventricular lesions showed the strongest anatomical gradient across groups. In contrast, infratentorial, juxtacortical and subcortical compartments were less discriminative and showed less consistent progression across diagnoses. This pattern is important because it suggests that not all regional WMH measures are equally informative for diagnostic stratification [46,47,48]. For a normative service intended for broad translational use, total lesion burden appears to offer the best compromise between robustness, interpretability, and reproducibility.

Another relevant aspect is the differing balance between calibration and discrimination in the 2D and 3D models. The 2D normative framework demonstrated moderate group separation, with higher Z-estimated values across the disease continuum and better overall ROC performance, whereas the 3D model showed fair-enough centering in cognitively normal individuals, with mean Z-scores closer to the theoretical expectation of zero. Importantly, a mean Z-estimated value in an external validation group is not always expected to be centered at zero, because the validation samples are independent from the reference population and often reflect different memory-clinic case mixes, demographic compositions, comorbidity profiles, and acquisition parameters. In this context, the fact that both models retained clinically meaningful normative information across distinct settings supports their practical value in real-world use. Taken together, our findings suggest that the 2D model is more sensitive to disease-related deviation, while the 3D model is better aligned with normative calibration. This is an informative property of the two modality-specific frameworks. Ideally, a clinically useful norm should balance both properties [37]: it should be sensitive enough to detect abnormality, but also sufficiently calibrated to avoid systematic inflation in healthy individuals. The availability of both 2D and 3D models therefore offers flexibility.

The ROC analyses reinforced this interpretation. Using GAMLSS-derived Z-scores, the 2D model achieved better overall discrimination than the 3D model in distinguishing cognitively normal individuals from pathological individuals, with a higher AUC and a threshold located at the upper tail of the normative distribution.

The secondary weighted-threshold analyses showed that the same models may be tuned for different clinical purposes. In particular, the 2D framework can be shifted toward a sensitivity-oriented operating point to support rule-out assessment, whereas the 3D framework can be shifted toward a specificity-oriented operating point to support rule-in confirmation. These weighted thresholds should be interpreted as application-driven operating points rather than universal cut-offs [49]. This complementary behavior is clinically meaningful.

All ROC-derived predictive values should be interpreted within the context of specialized secondary and tertiary memory-clinic cohorts, where the pre-test probability of cognitive impairment patients is substantially higher (ranging from 35 to 70%) [50,51,52] than in the general population (10–19%) [53,54]. In this setting, the pathological prevalence in the MCSA and NACC validation cohorts is consistent with that of memory clinics and with the intended use of the service as a second-opinion tool for patients undergoing evaluation for cognitive decline. Moreover, the service would be particularly suited to centers where MRI is already part of the diagnostic workup. The data support a sequential or context-dependent use of the two normative models. In practical terms, the 2D model may be especially valuable for wider clinical screening deployment, since 2D FLAIR is more commonly available and typically less costly than 3D acquisitions, more often associated with higher-field and more specialized protocols [55].

From a translational perspective, this work is interesting because it moves beyond the limitations of visual rating scales. Semi-quantitative scores such as Fazekas or Wahlund are useful in routine practice, but they remain subjective and are vulnerable to inter-rater variability [56,57]. In contrast, the proposed LST-AI-based framework produces standardized volumetric estimates and percentile scores that are more easily harmonized across centers and are suitable for second-opinion use, longitudinal monitoring, and large-scale research. Implementation within a publicly accessible online platform adds practical value, because it turns the norms into a service usable for researchers and clinicians without the need for local infrastructure or the purchase and complex configuration of algorithms for image processing.

The study has several strengths. The LST-AI enables performance improvements, particularly in the detection and segmentation of small lesions [10], compared to traditional tools [58]. The use of large normative samples enhances statistical stability and generalizability [59], while independent validation cohorts, modality-specific norms, and an advanced statistical framework for percentile derivation strengthen external validity. The validation cohorts were complementary, the 2D cohort provided a clinically heterogeneous but technically homogeneous setting, whereas the 3D cohort offered a multicenter and acquisition-heterogeneous scenario. This dual validation design increases confidence that the proposed framework is applicable to different imaging contexts.

Several limitations should be acknowledged. First, the validation cohorts are appropriate for external testing, but are not fully representative of all clinical settings, so the thresholds should not be generalized uncritically. Second, the 2D and 3D reference cohorts differed consistently with a high effect size in acquisition characteristics, particularly slice thickness and sequence-related time parameters, while sociodemographic variables likely contributed only partially to the observed differences between normative models. Third, partial availability of vascular risk factor data across validation cohorts may have introduced residual confounding. Fourth, visual quality control of LST-AI outputs was performed carefully, although no direct head-to-head comparison with visual WMH scales was conducted in the present work, limiting benchmarking of LST-AI against established clinical routines. Fifth, this work focused on cross-sectional discrimination and does not yet provide longitudinal evidence that the proposed cut-offs predict future cognitive decline, conversion, or WMH progression. Finally, while the operational and application-oriented thresholds defined are useful for classification, WMH burden is biologically and clinically continuous, so percentiles should complement, rather than replace, clinical judgment. These limitations do not diminish the contribution of the study. Instead, they delineate the boundaries within which the current framework should be interpreted: as a standardized, modality-aware, externally validated normative system for WMH quantification, rather than as general cut-offs applicable to all populations and protocols.

Future work should assess the transportability of these norms in broader clinical populations, evaluate their longitudinal prognostic value, and formally compare automated percentile-based interpretation with expert visual rating and other segmentation pipelines. Finally, we intend to integrate advanced harmonization techniques [60,61] to address multi-center scanner and field-strength heterogeneity in WMH quantification.

## 5. Conclusions

This work presents a practical and statistically grounded approach to interpreting LST-AI-derived WMH burden in individual subjects. By combining age- and gender-adjusted normative modeling, external validation, and deployment through the neuGRID platform, the study advances WMH quantification from a technical segmentation output to a clinically interpretable biomarker that may support diagnosis, stratification, and second-opinion assessment in cognitive disorders.

## Figures and Tables

**Table 1:** Normative population characteristics. Statistical comparisons were performed using the Mann–Whitney U test for continuous variables, with the false discovery rate according to the Benjamini–Hochberg correction (FDR-BH) applied for multiple comparisons, and the chi-square test for categorical variables. Effect-index, ranging from 0 to 1 and measuring association strength, was quantified using a standardized effect-size metric: for continuous variables, Cohen’s d was converted into effect-size correlation r-index; for categorical variables, association strength was estimated using Cramér’s V. Volumetric data are reported as median [Q1; Q3]. Symbols: ^(+)^ = chi-square test performed for the comparison; *: Quantile regression adjusted *p*-value. Acronyms: TE = Echo Time, TR = Repetition Time, TI = Inversion Time.

Normative Populations				
	2D	3D	*p*-Value	r-Index
Sample size, n	788	895		
Female, n (%)	453 (57.5)	544 (60.8)	*p* = 0.2	0.03
Age [years] Mean (StD)	66.4 (9.6)	70.8 (8.8)	*p* < 0.001	0.23
Education [years] Mean (StD)	15.1 (3.7)	17.1 (6.6)	*p* < 0.001	0.17
**Ethnicity, n (%)**	**2D**	**3D**	***p*-value**	**r-index**
White	708 (89.8)	655 (73.2)	*p* < 0.001 ^(+)^	0.24
Black	72 (9.1)	141 (15.8)		
Asian	6 (0.8)	46 (5.1)		
Other	2 (0.3)	53 (5.9)		
**Comorbidities, n (%)**	**2D**	**3D**	***p*-value**	**r-index**
Hypertension	175 (22.2)	86 (9.6)	*p* < 0.001 ^(+)^	0.23
Diabetes mellitus	36 (4.6)	34 (3.8)		
Heart disease	46 (5.8)	2 (0.2)		
Obesity	–	3 (0.3)		
**Scanner manufacturer, n (%)**	**2D**	**3D**	***p*-value**	**r-index**
Siemens	605 (76.8)	679 (75.9)	*p* < 0.001 ^(+)^	0.19
GE	172 (21.8)	127 (14.2)		
Philips	11 (1.4)	89 (9.9)		
**Flip angle, n (%)**	**2D**	**3D**	***p*-value**	**r-index**
150°	434 (55.1)	–	*p* < 0.001 ^(+)^	0.41
90°	183 (23.2)	216 (24.1)		
120°	132 (16.8)	669 (74.7)		
180°	38 (4.8)	10 (1.1)		
168°	1 (0.1)	–		
	**2D**	**3D**	***p*-value**	**r-index**
TE [s] Mean (StD)	0.1 (0.0)	0.4 (0.1)	*p* < 0.001	0.84
TR [s] Mean (StD)	8.5 (1.0)	5.0 (0.4)	*p* < 0.001	0.92
TI [s] Mean (StD)	2.3 (0.4)	1.7 (0.2)	*p* < 0.001	0.71
3T Field Strength, n (%)	545 (69.2)	895 (100)	*p* < 0.001 ^(+)^	0.44
Slice thickness [mm] Mean (StD)	4.97 (0.3)	1.1 (0.1)	*p* < 0.001	0.99
Infratentorial volume [mm^3^] Median [Q1; Q3]	4.7 [0.0; 61.4]	0 [0.0; 15.5]	*p* < 0.001 *	0.09
Juxtacortical lesion volume [mm^3^] Median [Q1; Q3]	193.2 [49.3; 630.9]	174.3 [36.8; 618.5]	*p* < 0.01 *	0.03
Periventricular lesion volume [mm^3^] Median [Q1; Q3]	317.0 [10.0; 1797]	728.8 [115.0; 3907.9]	*p* = 0.52 *	0.13
Subcortical volume [mm^3^] Median [Q1; Q3]	101.0 [16.4; 314.0]	194.8 [46.6; 547.3]	*p* < 0.05 *	0.13
Total lesion volume [mm^3^] Median [Q1; Q3]	935.7 [268.0; 3443.3]	1411.9 [386.0; 5604.3]	*p* = 0.37 *	0.11

**Table 2:** Characteristics of the independent 2D validation population stratified by diagnostic class. *p*-values were obtained with chi-square test for categorical and Kruskal–Wallis test for continuous variables. Post hoc analysis: º = significant difference between DM and CN, ^£^ = significant difference between DM and MCI, ^†^ = significant difference between MCI and CN. Symbols: ^(+)^ = chi-square test performed for the comparison; *: Quantile regression adjusted *p*-value. Acronyms: CN = Cognitive Normal Subject, MCI = Mild Cognitive Impairment, DM = Dementia Disease, MMSE = Mini-Mental State Examination, CDR-SB = Clinical Dementia Rating-Sum of Boxes, CDR= Clinical Dementia Rating, TE = Echo Time, TR = Repetition Time, TI = Inversion Time, s = seconds.

2D				
	CN	MCI	DM	*p*-Value
Z-estimated Mean (StD)	0.99 (0.75)	1.34 (0.74)	1.7 (0.5)	*p* < 0.05 º^,£,†^
Sample size, n	100	39	39	
Female, n (%)	50 (50.0)	18 (46.2)	13 (33.3)	*p* > 0.05
Age [years] Mean (StD)	70.7 (10.2)	70.4 (5.9)	81.9 (4.5)	*p* < 0.05 º^,£^
Education [years] Mean (StD)	15.1 (2.8)	13.5 (2.8)	13.1 (2.8)	*p* < 0.05 º^,†^
**Ethnicity, n (%)**	**CN**	**MCI**	**DM**	***p*-value**
White	100 (100)	38 (97.4)	37 (94.8)	*p* < 0.05 ^(+)^ º^,£,†^
Other	-	1 (2.6)	2 (5.2)	
**Comorbidities, n (%)**	**CN**	**MCI**	**DM**	***p*-value**
Hypertension	65 (65)	28 (71.8)	31 (79.5)	*p* > 0.05 ^(+)^
Diabetes mellitus	16 (16)	11 (28.2)	11 (28.2)	
**Scanner manufacturer, n (%)**	**CN**	**MCI**	**DM**	***p*-value**
GE	100 (100)	39 (100)	39 (100)	*p* = 1 ^(+)^ º^,£,†^
**Flip angle, n (%)**	**CN**	**MCI**	**DM**	***p*-value**
90°	100 (100)	39 (100)	39 (100)	*p* = 1 ^(+)^
	**CN**	**MCI**	**DM**	***p*-value**
TE [s] Mean (StD)	0.2 (0.0)	0.2 (0.0)	0.2 (0.0)	*p* > 0.05
TR [s] Mean (StD)	11.0 (0.0)	11.0 (0.0)	11.0 (0.0)	*p* > 0.05
TI [s] Mean (StD)	2.2 (0.0)	2.2 (0.0)	2.2 (0.0)	*p* > 0.05
3T Field Strength, n (%)	100 (100)	39 (100)	39 (100)	*p* = 1 ^(+)^ º^,£,†^
Slice thickness [mm] Mean (StD)	3.5 (0.2)	3.5 (0.2)	3.4 (0.3)	*p* < 0.05 ^(+),^º
Infratentorial volume [mm^3^] Median [Q1; Q3]	64.6 [8.5; 224.8]	90.0 [17.9; 244.9]	120.5 [2.1; 270.6]	*p* > 0.05 *
Juxtacortical lesion volume [mm^3^] Median [Q1; Q3]	1405.4 [424.3; 3233.4]	2298.6 [802.6; 4363.3]	2533.9 [1433.9; 6348.0]	*p* > 0.05 *
Periventricular lesion volume [mm^3^] Median [Q1; Q3]	2320.5 [692.6; 6758.2]	5354.0 [2075.1; 14,142.4]	16,472.8 [7477.9; 29,168.4]	*p* < 0.05 *^,^º^,£^
Subcortical volume [mm^3^] Median [Q1; Q3]	249.0 [83.7; 561.5]	372.0 [144.7; 782.4]	467.9 [360.2; 732.3]	*p* > 0.05 *
Total lesion volume [mm^3^] Median [Q1; Q3]	5141.5 [2063.8; 10,458.9]	10,394.4 [4699.7; 20,724.7]	21,210.8 [12,695.6; 32,137.1]	*p* < 0.05 *^,^º^,£^
CDR-SB Median [Q1; Q3])	0 [0; 0]	0 [0; 0.5]	4.5 [3; 5.50]	*p* < 0.05 *^,^º^,£,†^
CDR Median [Q1; Q3]	0 [0; 0]	0 [0; 0.5]	1 [0.5; 1]	*p* < 0.05 *^,^º^,£,†^
MMSE Median [Q1; Q3]	29 [28; 29]	26 [24; 27]	23 [21; 24]	*p* < 0.05 *^,^º^,£,†^

**Table 3:** Characteristics of the independent 3D validation population stratified by diagnostic class. *p*-values were obtained with chi-square test for categorical and Kruskal–Wallis test for continuous variables. Post hoc analysis: º = significant difference between AD and CN, ^£^ = significant difference between AD and MCI, ^†^ = significant difference between MCI and CN. Symbols: ^(+)^ = chi-square test performed for the comparison; *: Quantile regression adjusted *p*-value. Acronyms: CN = Cognitive Normal Subject, MCI = Mild Cognitive Impairment, AD = Alzheimer Disease, MoCA = Montreal Cognitive Assessment, CDR-SB = Clinical Dementia Rating-Sum of Boxes, TMT-A = Trail Making Test Part A and TMT-B = Trail Making Test Part B, TE = Echo Time, TR = Repetition Time, TI = Inversion Time, s = seconds.

3D				
	CN	MCI	AD	*p*-Value
Z-estimated Mean (StD)	−0.08 (0.97)	0.37 (1.12)	0.71 (1.17)	*p* < 0.05 º^,£,†^
Sample size, n	90	95	95	*p* > 0.05
Female, n (%)	46 (51.1)	49 (51.6)	49 (51.6)	*p* > 0.05
Age [years] Mean (StD)	69.2 (10.4)	71.8 (8.0)	70.0 (9.9)	*p* > 0.05
Education [years] Mean (StD)	16.1 (2.6)	16.4 (2.6)	15.7 (2.6)	*p* > 0.05
**Ethnicity, n (%)**	**CN**	**MCI**	**AD**	***p*-value**
White	79 (87.7)	79 (83.2)	88 (92.6)	*p* > 0.05 ^(+)^
Black	10 (11.1)	12 (12.6)	6 (6.3)	
Other	1 (1.1)	2 (2.1)	-	
**Comorbidities, n (%)**	**CN**	**MCI**	**AD**	***p*-value**
Hypertension	14 (15.5)	23 (24.2)	20 (21.0)	*p* > 0.05 ^(+)^
Diabetes mellitus	5 (5.5)	7 (7.4)	7 (7.4)	
Heart Disease	-	-	1 (1.1)	
**Scanner manufacturer, n (%)**	**CN**	**MCI**	**AD**	***p*-value**
Siemens	49 (54.4)	59 (62.1)	75 (78.9)	*p* < 0.05 ^(+),^º
GE	18 (20.0)	13 (13.7)	6 (6.3)	
Philips	23 (25.6)	23 (24.2)	14 (14.7)	
**Flip angle, n (%)**	**CN**	**MCI**	**AD**	***p*-value**
90°	41 (45.5)	36 (37.9)	20 (21.0)	*p* < 0.05 ^(+),^º
120°	49 (54.5)	59 (62.1)	75 (79.0)	
	**CN**	**MCI**	**AD**	***p*-value**
TE [s] Mean (StD)	0.3 (0.1)	0.4 (0.1)	0.4 (0.1)	*p* > 0.05
TR [s] Mean (StD)	4.8 (0.1)	4.8 (0.1)	4.9 (0.1)	*p* < 0.05 º^,£^
TI [s] Mean (StD)	1.6 (0.1)	1.6 (0.1)	1.6 (0.1)	*p* > 0.05
3T Field Strength, n (%)	90 (100)	95 (100)	95 (100)	*p* = 1 ^(+)^ º^,£,†^
Slice thickness [mm] Mean (StD)	1.2 (0.1)	1.2 (0.1)	1.1 (0.1)	*p* < 0.05 ^(+),^º^,£^
Infratentorial volume [mm^3^] Median [Q1; Q3]	0.0 [0.0; 7.2]	0.0 [0.0; 54.8]	0.0 [0.0; 14.4]	*p* > 0.05 *
Juxtacortical lesion volume [mm^3^] Median [Q1; Q3]	102.2 [13.4; 246.9]	197.9 [65.5; 1112.6]	192.1 [30.5; 909.5]	*p* > 0.05 *
Periventricular lesion volume [mm^3^] Median [Q1; Q3]	519.5 [110.8; 2017.1]	1124.0 [202.1; 9150.8]	3062.2 [1202.6; 11,356.3]	*p* < 0.05 *^,^º^,£^
Subcortical volume [mm^3^] Median [Q1; Q3]	109.1 [24.6; 319.0]	252.0 [71.2; 594.5]	208.2 [59.5; 506.9]	*p* > 0.05 *
Total lesion volume [mm^3^] Median [Q1; Q3]	1033.5 [232.4; 2694.2]	2225.1 [620.4; 11,213.8]	4438.3 [1558.9; 14,731.9]	*p* < 0.05 *^,^º^,£^
CDR-SB Median [Q1; Q3]	0 [0; 0]	1 [0.5; 1.5]	5 [4; 9]	*p* < 0.05 *^,^º^,£,†^
TMT-A Median [Q1; Q3]	26 [21; 33]	34.5 [29; 46]	50 [35; 92.25]	*p* < 0.05 *^,^º^,£,†^
TMT-B Median [Q1; Q3]	67 [53; 83.75]	101 [75.25; 146]	176.5 [95.25; 300]	*p* < 0.05 *^,^º^,£,†^
MoCA Median [Q1; Q3]	27 [25; 28]	23 [20; 26]	15 [9; 18]	*p* < 0.05 *^,^º^,£,†^

**Table 4:** Diagnostic accuracy was assessed using AUC as the primary discrimination metric with optimal and weighted cut-offs (maximizing LR− in rule-out for 2D normative model, and LR+ in rule-in for 3D normative model). Acronyms: CN = cognitive normal, MCI = mild cognitive impairment, AD = Alzheimer’s Dementia, DM = Dementia, AUC = Area Under the Curve, CI = Confidence Interval, PPV = Positive Predictive Value, NPV = Negative Predictive Value, LR+ = positive likelihood ratio, LR− = negative likelihood ratio.

Normative Model	Comparison	Weight	AUC [95% CI]	Threshold	Sensitivity	Specificity	PPV	NPV	LR+	LR−
2D	CN vs. (MCI + DM)	Standard (J = 0.5)	0.71 [0.63–0.78]	1.438 (92nd percentile)	0.62	0.71	0.62	0.70	2.12	0.54
		Weighted (J = 0.65)	0.71 [0.63–0.78]	0.494 (69th percentile)	0.95	0.26	0.5	0.87	1.28	0.20
3D	CN vs. (MCI + AD)	Standard (J = 0.5)	0.68 [0.61–0.74]	1.025 (85th percentile)	0.43	0.90	0.90	0.43	4.32	0.63
		Weighted (J = 0.25)	0.68 [0.61–0.74]	1.381 (92nd percentile)	0.28	0.97	0.95	0.39	8.53	0.74

## Data Availability

The original data presented in the study are openly available in NeuGrid at https://neugrid2.eu (accessed on 14 June 2026), in IDA at https://ida.loni.usc.edu/ (accessed on 14 June 2026) and in OASIS at https://sites.wustl.edu/oasisbrains/ (accessed on 14 June 2026).

## Supplementary Materials

The following supporting information can be downloaded at: https://www.mdpi.com/article/10.3390/diagnostics16152460/s1.
