# Supplementary material for: Norms for Automatic Estimation of White Matter Hyperintensities Burden: LST-AI Service in neuGRID
Source: Diagnostics (Basel). 2026 Aug 4;16(15):2460. doi: 10.3390/diagnostics16152460 (PMC13464935; doi:10.3390/diagnostics16152460)
Supplement: Supplementary file 1 [file diagnostics-16-02460-s001.zip › diagnostics-4409666-supplementary.pdf]

SUPPLEMENTARY MATERIALS

1. Explained Variance Score for 2D and 3D norms

Percentage of variance explained for each feature.

Region	Covariate	Variance (%) in 2D norms	Variance (%) in 3D norms
**Infratentorial**	age	0	0
	sex	0	0
	education	0	0
	ethnicity	0.21	0
	comorbidities	0	0.17
	manufacturer	0.5	0
	field strength	0.85	0
	TE	0	0
	TR	0	0
	TI	0.06	0
	flip angle	0	0
	slice thickness	0.17	0
**Juxtacortical**	age	0.75	1.84
	sex	0.19	0.88
	education	0	0.11
	ethnicity	0.05	0.05
	comorbidities	0.08	0.14
	manufacturer	0	1.66
	field strength	0	0
	TE	0.07	0.1
	TR	0	0.33
	TI	0	0.11
	flip angle	0	2.89
	slice thickness	0	0.21
**Periventricular**	age	2.87	6.82
	sex	0	0
	education	0.12	0
	ethnicity	0.53	0
	comorbidities	1.7	0

	manufacturer	0.09	0
	field strength	0.09	0.01
	TE	0	0.06
	TR	0	0
	TI	0	0
	flip angle	0.12	0.12
	slice thickness	0.30	0
**Subcortical**	age	4.84	8.54
	sex	0.51	0.09
	education	0	0.02
	ethnicity	0.6	0
	comorbidities	0.54	0.87
	manufacturer	0	0.33
	field strength	0	0
	TE	0.26	0.07
	TR	0	0.21
	TI	0.16	0
	flip angle	0	1.75
	slice thickness	0.75	0.3
**Total**	age	4.58	8.58
	sex	0	0.18
	education	0	0
	ethnicity	0.47	0
	comorbidities	1.5	0
	manufacturer	0	0
	field strength	0	0
	TE	0	0.18
	TR	0	0
	TI	0	0
	flip angle	0	0.29
	slice thickness	0.31	0.11

Supplementary Table S1. Explained variance of the model features on WMH total volumes in the normative datasets using quantile regression (q=0.5). The unique proportion of total variance explained by each individual covariate was quantified using a drop-variable approach. Specifically, the semi-partial explained variance was computed as the difference in the Explained Variance Score between the full model and the reduced model excluding the specific variable of interest.

2. Adjusted predicted medians for 2D and 3D norms

Due to the right-skewness of WMH lesion volume, associations were examined using median quantile regression. Normative models (2D vs 3D) were adjusted for age, sex, ethnicity, and years of education. The coefficients were interpreted as additive shifts in the conditional median total WMH volume. Adjusted median estimates and between-group contrasts were derived from model predictions, with uncertainty assessed by bootstrap resampling. Pairwise contrasts on adjusted medians are reported in Supplementary Table 2a and 2b. Expected 2D and 3D conditional medians for typical subject profiles is reported in Supplementary Table 2c and each covariate represents an additive shift of the conditional median as reported in Supplementary Table 2d.

**Group**	**Adjusted predicted median**	**SE**	**95% CI**	**N° boostrap used**
2D Normative group	1828.4	148.9	[1561.4; 2155.6]	1000
3D Normative group	1704.9	128.1	[1497.1; 1975.6]	1000

Supplementary Table S2a: Adjusted and standardized predicted medians by normative group.

**Contrast**	**Estimate median difference**	**SE**	**95% CI**	**P-value**	**FDR-BH**
2D - 3D	127.4	141.4	[-130.3; 413.4]	0.37	0.37

Supplementary Table S2b: Adjusted median difference between 2D and 3D groups.

**Group**	**Age**	**Sex**	**Ethnicity**	**Education**	**Predicted conditional median**
2D Normative group	55	F	W	16	212.2
2D Normative group	65	F	W	16	1372.8
2D Normative group	75	F	W	16	2533.4
2D Normative group	85	F	W	16	3693.9
3D Normative group	55	F	W	16	93.8
3D Normative group	65	F	W	16	1254.4
3D Normative group	75	F	W	16	2415.0
3D Normative group	85	F	W	16	3575.6

Supplementary Table S2c: Prediction grid for conditional medians.

**Term**	**Beta median shift**	**95% CI**	**P-value**
(Group, Treatment(reference='2D'))[T.3D]	-118.3	[-377.7; 2155.6]	0.37
(sex)[T.M]	-125.7	[-373.5; 122.1]	0.32
(ethnicity)[T.Black]	464.2	[-290.9; 1219.4]	0.23
(ethnicity)[T.Other]	537.2	[-416.3; 1490.8]	0.27
(ethnicity)[T.White]	405.7	[-288.9; 1100.5]	0.25
age	116.1	[102.7; 129.4]	0.00
education	-6.5	[-28.5; 15.5]	0.56

Supplementary Table S2d: Median shift quantified for each coefficient, 2D group is used as reference.

3. WMH lesions in the independent cohort

Lesion Percentile Progression (2D - MSCA)			
**Lesion Percentile - Region**	**CN**	**MCI**	**DM**
**Infratentorial** *Median [Q1 Q3]*	89.5 [58.7 96.7]	92.2 [70.9 97.0]	92.8 [22.1 97.1]
**Juxtacortical** *Median [Q1 Q3]*	88.6 [73.0 95.5]	93.3 [82.2 97.1]	92.9 [86.7 97.6]
**Periventricular** *Median [Q1 Q3]*	90.2 [76.8 96.4]	95.0 [88.3 98.4]	98.2 [95.7 99.1]
**Subcortical** *Median [Q1 Q3]*	77.2 [56.6 88.5]	84.9 [68.9 92.5]	81.2 [77.1 87.5]
**Total** *Median [Q1 Q3]*	85.5 [68.1 94.1]	93.1 [82.1 97.3]	96.1 [91.8 97.5]

Lesion Percentile Progression (3D - NACC)			
**Lesion Percentile - Region**	**CN**	**MCI**	**AD**
**Infratentorial** *Median [Q1 Q3]*	0.1 [0.0 88.7]	73.0 [0.0 98.6]	2.3 [0.0 93.7]
**Juxtacortical** *Median [Q1 Q3]*	50.3 [17.5 68.6]	60.9 [44.7 87.8]	67.3 [26.2 86.2]
**Periventricular** *Median [Q1 Q3]*	58.0 [29.8 82.2]	68.5 [33.9 94.2]	89.1 [75.9 96.4]
**Subcortical** *Median [Q1 Q3]*	48.3 [16.0 71.8]	62.4 [30.2 80.8]	57.8 [34.1 77.5]
**Total** *Median [Q1 Q3]*	47.6 [27.2 73.5]	64.8 [31.9 91.2]	84.4 [62.3 93.3]

Supplementary Table S3. Progressive distribution of lesion volumes and corresponding percentiles relative to the 3D and 2D normative datasets across diagnostic groups in the 2D and 3D validation population. Acronyms: ***:*** *CN = Cognitive Normal Subject, MCI = Mild Cognitive Impairment, AD = Alzheimer Disease, DM = Dementia Disease.*

2.1 NeuGRID web-portal

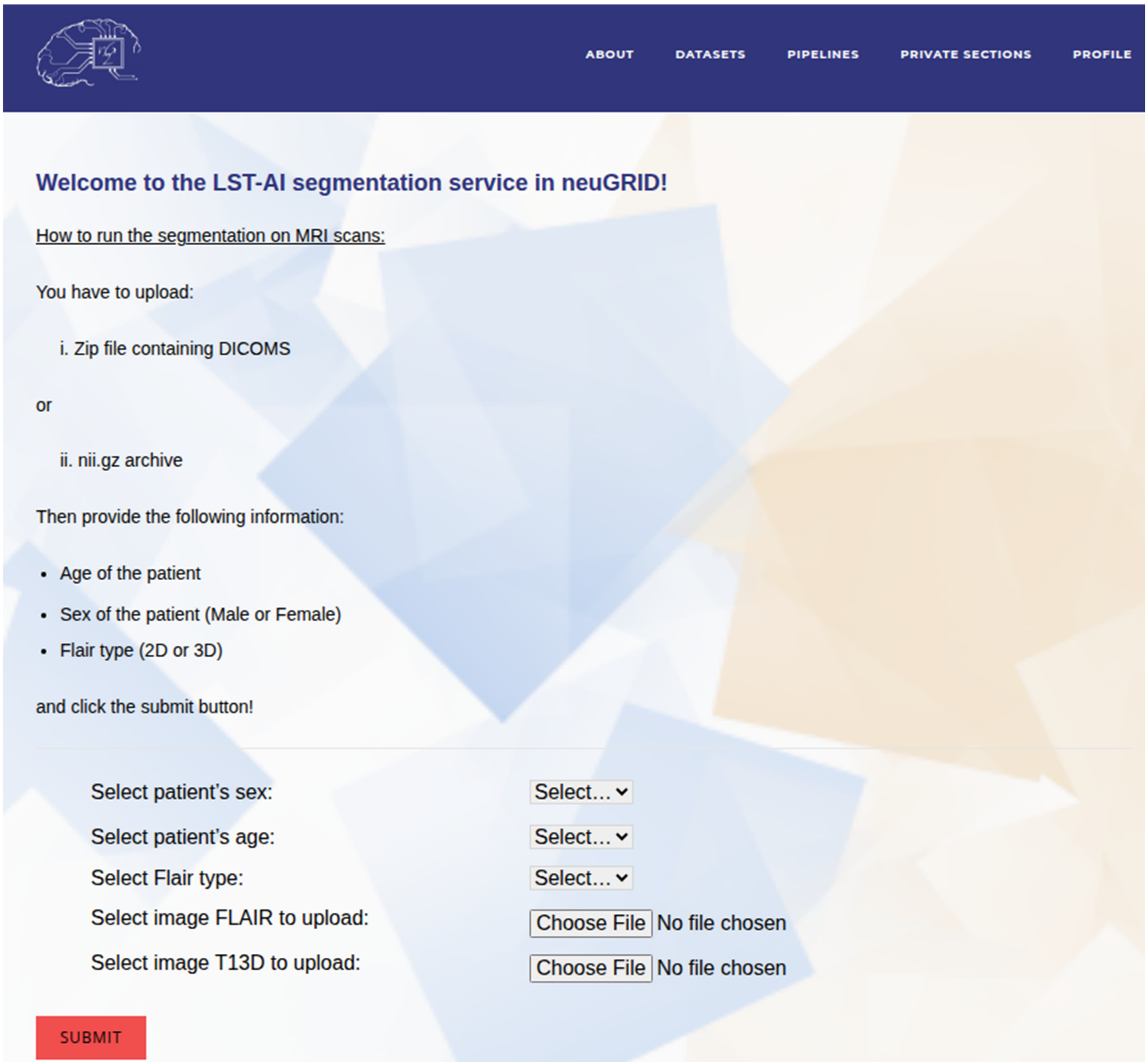

Supplementary Figure S1: Interface of the LST-AI pipeline in the neuGRID platform. The end-user must enter the information required by the pipeline (i.e.: age, gender, FLAIR typology (2D or 3D)), FLAIR and T13D scans. The LST-AI pipeline runs on the neuGRID High Performance Computing centre (https://neugrid2.eu) to produce a PDF report (see Supplementary Figure 2).

2.2 LST-AI Report

LST-AI Report

Subject info

- Patient ID: **LST-AI-FL-FLAIR-native**
- Sex: **M**
- Age: **85**

LST-AI Results

**Region**	**Volume [mm^3]**	**Percentile**	**State**
Infratentorial	69.6	–	–
Juxtacortical	254.6	–	–
Periventricular	1275.0	–	–
Subcortical	515.0	–	–
TOTAL	2114.2	44	non-pathological

Table 1: Volumes (mm^3) of white matter lesions in 5 analysed ROIs. The percentile of single subject is derived by comparing each ROI to 895 healthy controls. The STATE column reports if the subject is pathological (above 85th percentile) or not (below 85th percentile).

LST-AI WM Segmentation[1]

• Total

Total Lesion Volume: 2114.2 mm^3 | Total Lesion Number: 84.0

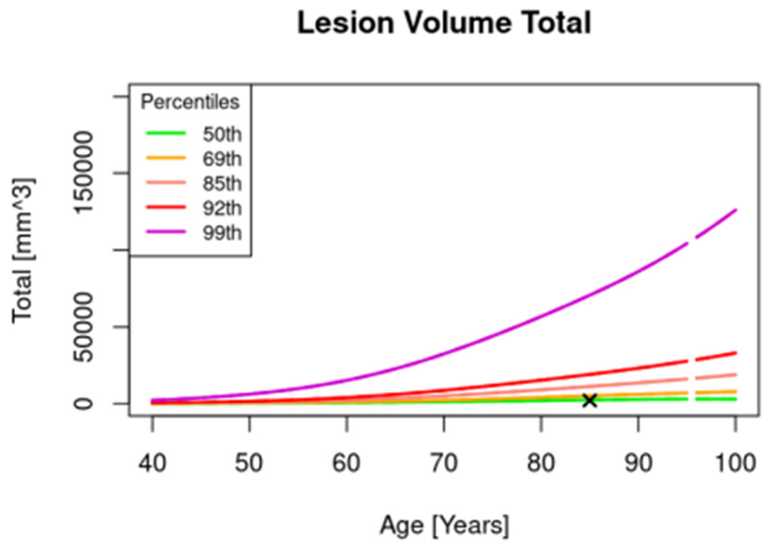

[1]Each coloured line represents the percentile for white matter lesion volume derived from non-pathological subjects across multiple normative datasets, as reported in Boccali et al., 2026 (under preparation)

Supplementary Figure S2: Example of LST-AI report sent as PDF to the end-user's mailbox and generated via neuGRID (https://neugrid2.eu). The report includes the value and graphical representation of LST-AI features organized into three main sections (i.e.: Subject info, Results and Segmentation).
